# In Vitro and In Vivo Pharmacological Characterization of a Novel TRPM8 Inhibitor Chemotype Identified by Small-Scale Preclinical Screening

**DOI:** 10.3390/ijms23042070

**Published:** 2022-02-13

**Authors:** Nunzio Iraci, Carmine Ostacolo, Alicia Medina-Peris, Tania Ciaglia, Anton M. Novoselov, Andrea Altieri, David Cabañero, Asia Fernandez-Carvajal, Pietro Campiglia, Isabel Gomez-Monterrey, Alessia Bertamino, Alexander V. Kurkin

**Affiliations:** 1 Department of Chemical, Biological, Pharmaceutical and Environmental Sciences, University of Messina, Viale Ferdinando Stagno d’Alcontres 31, 98166 Messina, Italy; nunzio.iraci@unime.it; 2 Department of Pharmacy, University of Naples Federico II, Via D. Montesano 49, 80131 Naples, Italy; ostacolo@unina.it (C.O.); imgomez@unina.it (I.G.-M.); 3 Instituto de Investigación, Desarrollo e Innovación en Biotecnología Sanitaria de Elche (IDiBE), Universidad Miguel Hernández de Elche, Avenida de la Universidad, 03202 Elche, Spain; alicia.medinap@umh.es (A.M.-P.); dcabanero@umh.es (D.C.); asia.fernandez@umh.es (A.F.-C.); 4 Department of Pharmacy, University of Salerno, Via G. Paolo II, 84084 Fisciano, Italy; tciaglia@unisa.it (T.C.); pcampiglia@unisa.it (P.C.); 5 Department of Chemistry, Lomonosov Moscow State University, 1/3 Leninsky Gory, 119991 Moscow, Russia; antonnowoselov@yandex.ru (A.M.N.); aaltieri@edasascientific.com (A.A.); 6 EDASA Scientific srls, Via Stingi 37, 66050 San Salvo, Italy

**Keywords:** TRPM8 antagonist, virtual screening, calcium fluorimetry, in silico studies, patch-clamp electrophysiology, oxaliplatin-induced allodynia model

## Abstract

Transient receptor potential melastatin type 8 (TRPM8) is a target for the treatment of different physio-pathological processes. While TRPM8 antagonists are reported as potential drugs for pain, cancer, and inflammation, to date only a limited number of chemotypes have been investigated and thus a limited number of compounds have reached clinical trials. Hence there is high value in searching for new TRPM8 antagonistic to broaden clues to structure-activity relationships, improve pharmacological properties and explore underlying molecular mechanisms. To address this, the EDASA Scientific in-house molecular library has been screened in silico, leading to identifying twenty-one potentially antagonist compounds of TRPM8. Calcium fluorometric assays were used to validate the in-silico hypothesis and assess compound selectivity. Four compounds were identified as selective TRPM8 antagonists, of which two were dual-acting TRPM8/TRPV1 modulators. The most potent TRPM8 antagonists (**BB 0322703** and **BB 0322720**) underwent molecular modelling studies to highlight key structural features responsible for drug–protein interaction. The two compounds were also investigated by patch-clamp assays, confirming low micromolar potencies. The most potent compound (**BB 0322703**, IC_50_ 1.25 ± 0.26 μM) was then profiled in vivo in a cold allodinya model, showing pharmacological efficacy at 30 μM dose. The new chemotypes identified showed remarkable pharmacological properties paving the way to further investigations for drug discovery and pharmacological purposes.

## 1. Introduction

Transient receptor potential melastatin type 8 (TRPM8) is emerging as a potential target for the treatment of a variety of disorders, given its key role in many physio-pathological processes [1]. It is a non-selective Ca^2+^ permeable channel that shows a highly complicated gating behaviour regulated by multiple factors [2,3] including temperature, endogenous and exogenous modulators or mechanisms, and depolarizing voltages [4,5]. 

TRPM8 is expressed mainly in a subpopulation of primary afferent neurons present in dorsal root and trigeminal ganglia, and in the nodose and geniculate ganglia of the peripheral nervous system. In these neurons, TRPM8 participates in the amplification of pain sensation after injury [6], suggesting that TRPM8 channel blockers could be effective for the treatment of chronic pain and migraine [7,8]. Furthermore, TRPM8 modulators have been investigated as therapeutics for the symptomatology of bladder pain syndromes, given their increased expression in patients suffering from these disorders [9]. Moreover, the channel overexpression in different kinds of tumors, such as prostate tumor [10], melanoma [11], breast cancer [12], and human pancreatic adenocarcinoma [13], together with the evidence of its implication in cellular proliferation, survival, and invasion, makes it an attractive target for cancer modulation [14]. The critical role of this receptor in humans and all living beings [15,16,17] has also been witnessed by the 2021 Nobel prize in Physiology and Medicine [18]. 

Despite the high interest in this area, to date, the number of known TRPM8 modulators is limited to a few natural agonists, such as menthol, or synthetic derivatives, like icilin [19,20] and a few synthetic and natural antagonists, including BCTC, CTPC, and capsazepine [21]. Of note, the antifungal drug clotrimazole has strong TRPM8 antagonistic activity [22], while SKF96365, a non-specific blocker of several types of calcium channels, also inhibits TRPM8 in vitro [23]. Finally, a cyclic peptide specifically targeting the outer pore of the channels has been described as a potent antagonist endowed with anti-allodynic effects [24]. Nevertheless, none of the described TRPM8 antagonists has reached the clinical stage, so far [25]. Thus, the discovery of new chemotypes acting as TRPM8 antagonists appears important in order to improve the knowledge about their structure-activity relationships, to reduce some of the reported side effects [26], and to further investigate the molecular mechanisms underlying TRPM8 antagonism. Our research group has been deeply involved in the design and synthesis of new TRPM8 modulators. In particular, we have identified several chemotypes, showing a very remarkable inhibitory effect against TRPM8 channels and selectivity over other TRPs [27,28,29]. Some of these derivatives exhibited in vivo analgesic activity [30], while other compounds were characterized for their inhibitory effect over androgen-dependent prostate cancer cell proliferation, migration, and invasiveness [31]. On the other hand, structure-based virtual screening represents an outstanding alternative tool for putative hit compounds discovery, allowing the investigation of large chemical libraries through computational methods by applying knowledge about the protein target [32]. This approach has been profitably used for the identification of several chemotypes as TRPM8 antagonists [25,33]. 

In this work, starting from the recently released cryo-EM structure of the antagonist-bound *Parus major* TRPM8 [2], we have built a homology model of the human TRPM8 binding site. Then, a virtual screening (VS) protocol was applied to a commercially available molecular library (http://zinc12.docking.org/catalogs/edasa/targets/clustered, lastly accessed on 31 December 2021), leading to the identification of two novel chemotypes as potent and selective inhibitors of menthol-evoked TRPM8 currents, as assessed by Ca^2+^-microfluorometric imaging and patch-clamp assays. The effect of one of these compounds was also studied in an in vivo model of cold allodynia, revealing its efficacy and suitability for further development.

## 2. Results

### 2.1. Virtual Screening

In order to maximize the chances to identify active inhibitors by testing just a small number of molecules, the library was submitted to a docking-based virtual screening (VS) workflow using a two targets ensemble. In particular, we used two homology models of human TRPM8 (hTRPM8) domains S1 to TRP (residues 723–1013) in homotetrameric assembly. The models were built by homology modelling using as templates the Cryo-EM structures of Parus major TRPM8 in complex with AMTB (PDB ID: 6O6R) and TC-I 2014 (PDB ID: 6O72) [2]. The whole library was docked into the two target structures using a stepwise protocol consisting of two docking stages. Compounds were initially flexibly docked using Glide SP, and the best scoring ones were advanced to the following step that refined and rescored the docking poses using Glide XP (see materials and methods), finally giving a list of compounds ranked according to their XP GlideScore values, that ranged from −11.982 to −8.903. Twenty-one compounds were selected (Table 1) from the list of top-scoring 100 compounds (Table S1) for the following experimental screening, on the basis of their stock availability and quality control results.

### 2.2. Screening by Ca^2+^-Microfluorometric Assay

**Figure 1:**
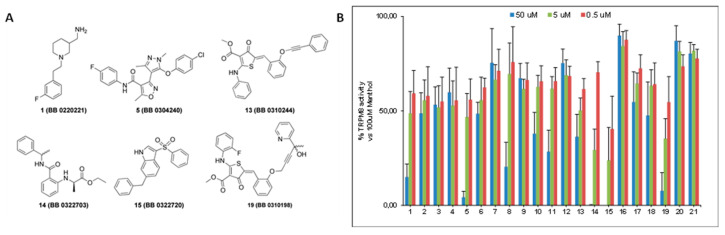
(**A**) Molecular structures of the most active compounds as TRPM8 blockers in Calcium fluorometric assays. In brackets are the original library codes (**B**) Bar-graph reporting the blocking activity of the 21 compounds arising from the virtual screening protocol against TRPM8 menthol-evoked currents.

The inhibitory activity of the 21 selected compounds over TRPM8 was challenged by Ca^2+^ fluorometric assays using HEK293 cells stably expressing the rat isoform of TRPM8 channels. Menthol and AMTB were used as reference agonist and antagonist, respectively. The compounds were initially tested at three different concentrations (50 μM, 5 μM, and 0.5 μM) to assess their ability in blocking 100 μM menthol evoked currents. As shown in Figure 1, six out of twenty-one compounds (**1**, **5**, **13**–**15**, and **19**) showed a remarkable antagonist activity.

Calculation of the IC_50s_ for these compounds led to the identification of TRPM8 inhibitors with a potency that was comparable or higher than the canonical TRPM8 antagonist AMTB [27], in the 11.1–0.22 μM range (Table 2). Moreover, two compounds, namely **14 (BB 0322703)** and **15 (BB 0322720)**, together with the lowest IC_50_ among this set of derivatives (0.25 and 0.22 μM respectively), showed 100% of efficacy in TRPM8 blocking.

Results obtained for the prototypical TRPM8 blocker AMTB are in accordance with the literature [27]. Among the selected compounds **1**, **5,** and **19** showed comparable potencies to AMTB. On the other hand, compound **13** showed a 7-fold decrease in potency, maintaining 90% of efficacy. Compounds **14** and **15** were the most promising compounds of the series. They both showed 100% efficacy in blocking the menthol-evoked currents, with IC_50s_ about 30-folds lower than the reference compound AMTB, comparable with the calcium fluorometric results obtained for the most potent in vitro TRPM8 inhibitor described so far [25,26,27]. 

### 2.3. Molecular Modeling

**Figure 2:**
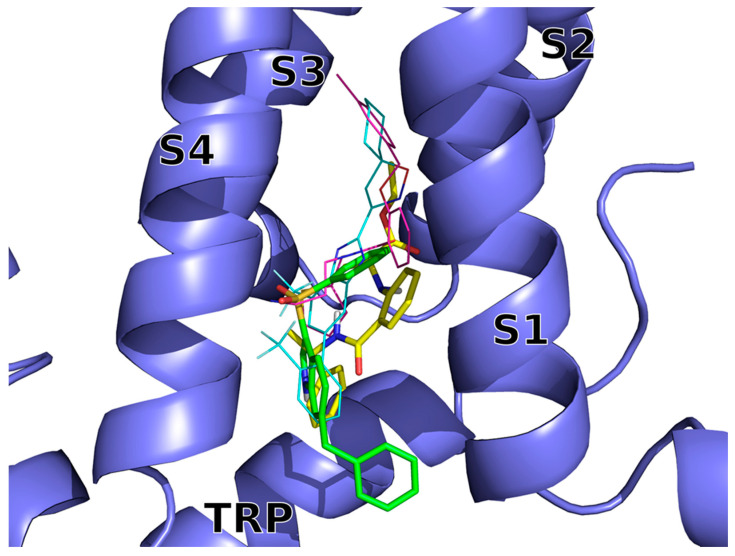
Docking-predicted bound conformations of **14** (**BB 0322703**) and **15** (**BB 0322720**) into hTRPM8_723–1013_. hTRPM8 is represented in blue cartoons. **14** (**BB 0322703**) and **15** (**BB 0322720**) in their hTRPM8-bound conformations are represented in yellow and green thick sticks, respectively. TC-I 2014 (PDB ID: 6O72) and AMTB (PDB ID: 6O6R) experimental bound conformations are shown in cyan and magenta thin sticks, respectively, for reference.

The VS docking poses of compounds **14** (**BB 0322703**) and **15** (**BB 0322720**) (Figure 2) were submitted to molecular dynamics (MD) simulations in explicit solvent and membrane to get insight into the ligand/hTRPM8 interactions. 

The two inhibitors dock into the VSLD (voltage-sensor-like domain) assuming similar bound conformations, surrounded by S1–S4 and TRP residues, with both of them lacking interactions with the “roof” of the binding site, that is instead exploited by AMTB and TC-I 2014 (Figure 2).

**Figure 3:**
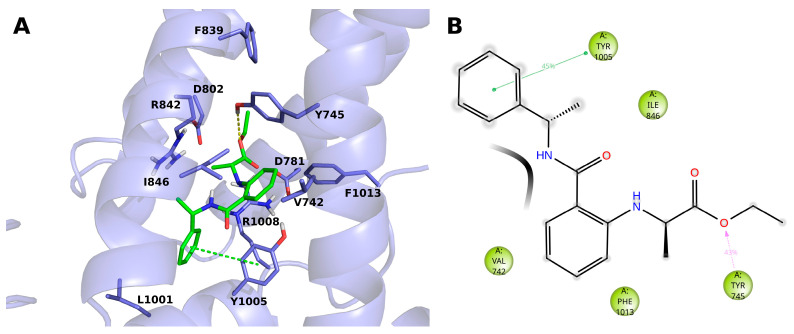
(**A**) Docking-predicted pose of **14** (**BB 0322703**) into hTRPM8_723–1013_. hTRPM8 is represented in cartoons, and its residues interacting with the ligand are represented in sticks. **14** (**BB 0322703**) in its hTRPM8-bound conformation is represented in solid green sticks. H-bonds are highlighted by yellow dashed lines, π–π interaction by green dashed lines. (**B**) Ligand interaction diagram of **14 (BB 0322703**) in complex with hTRPM8. Only residues interacting with the ligand for at least 40 ns out 120 ns of MD simulation are shown. Hydrophobic residues are colored green. Grey halos highlight solvent exposure. H-bonds are represented by magenta arrows (dashed when side-chain atoms are involved); green solid lines represent π–π interactions.

The compound **14 (BB 0322703**) docks into the VSLD, where it stably interacts with residues V742, Y745, I846, Y1005, and F1013. (Figure 3A,B). The terminal phenyl moiety π-π stacks with Y1005, while another tyrosine residue, i.e., 745, interacts with the ligand ester oxygen through a hydrogen bond. Tyrosine 745 interacts, although to a lesser extent, by π-π stacking with the ligand core phenyl ring.

**Figure 4:**
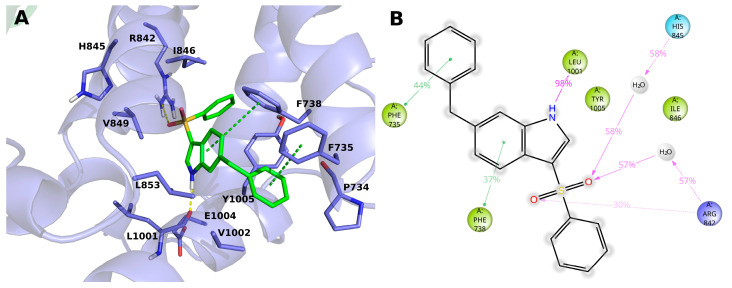
(**A**) Docking-predicted pose of **15** (**BB 0322720**) into hTRPM8_723–1013_. hTRPM8 is represented in cartoons, and its residues interacting with the ligand are represented in sticks. **15** (**BB 0322720**) in its hTRPM8-bound conformation is represented in solid green sticks. H-bonds are highlighted by yellow dashed lines, π–π interaction by green dashed lines. (**B**) Ligand interaction diagram of **15** (**BB 0322720**) in complex with hTRPM8. Only residues interacting with the ligand for at least 40ns out 120ns of MD simulation are shown. Residues are coloured according to the following scheme: cyan—polar; blue—charged (positive); green—hydrophobic; gray—water molecule. Grey halos highlight solvent exposure. H-bonds are represented by magenta arrows (dashed when side-chain atoms are involved); green solid lines represent π–π interactions.

The compound **15** (**BB 0322720**) docks in the lower part of the VSLD, on top of the TRP box, where it stably interacts with several residues. (Figure 4A,B) In particular, the benzyl moiety sticks out of VSLD, in a region surrounded by hydrophobic residues P734, V1002, Y1005, and F735. The latter phenylalanine residue stably makes π–π interaction with the aromatic group. The indole ring, surrounded by residues V849, L853, L1001, V1002, E1004, Y1005, F735, and F738, makes an H-bond with the backbone of L1001 by its nitrogen atom, and π-π interacts with F738. The benzenesulfonyl moiety is surrounded by residues I846, Y1005, F738, R1008, and R842, and its sulfonyl oxygens make both direct and water-mediated H-bonds with H845 and R842. It is worth noting that, in our model, the phenyl group does not make any specific and stable interaction during the MD simulation time but offers a handle to grow the molecule toward the VSLD roof, where additional interaction with residues such as Y745, L778, L806, F839, D802, Q782, D781 might be established, as in the case of AMTB, TC-I-2014, and many other TRPM8 modulators.

### 2.4. Selectivity Studies

**Figure 5:**
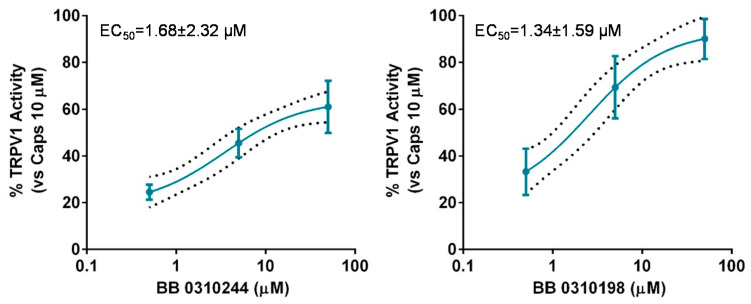
Dose-response curve for **13** (**BB 0310244**) (**left panel**) and **19** (**BB 0310198**) (**right panel**) for TRPV1 activation. Response is expressed as percentage of TRPV1 activation normalized to the effect of the prototypical agonist capsaicine at 10 μM, considered as 100% of activation. The discontinuous line shows the 95% confidence intervals. The solid line represents fits of the experimental data to the following equation: Y = Bottom + (Top − Bottom)/(1 + 10^((LogEC50-X)^^×^^HillSlope)^), with the restriction of the minimum (Bottom = 0).EC50 = 1.69 (0.26–10.73) μM with r*^2^*= 0.82 and Hill Slope = 0.53 for **13** (**BB 0310244**) and EC50 = 1.34 (0.49–3.62) μM with r*^2^* = 0.85 and Hill Slope = 0.67 for **19** (**BB 0310198**).

The lack of selectivity of TRPM8 modulators over other members of the TRP channel family is widely reported in literature [25,26,27]. Cross modulation of TRPV1 by prototypical TRPM8 agonists and antagonists, such as menthol [34] and BCTC [35] is reported and reciprocal modulation of these two channels has been proposed as suitable strategy to attain synergistic pharmacological effects [36,37,38]. Therefore, we decided to screen the library members showing TRMP8 inhibition over TRPV1 using calcium-fluorometric experiments. None of the compounds proved modulatory ability over TRPV1 either as activators or inhibitors (data not shown), with the exceptions of compounds **13** (**BB 0310244**) and **19** (**BB 0310198**) showing low micromolar potencies as TRPV1 agonists, with 1.34 < EC_50s_ < 1.68 μM (Figure 5). 

### 2.5. Patch-Clamp Electrophysiology Assays

**Figure 6:**
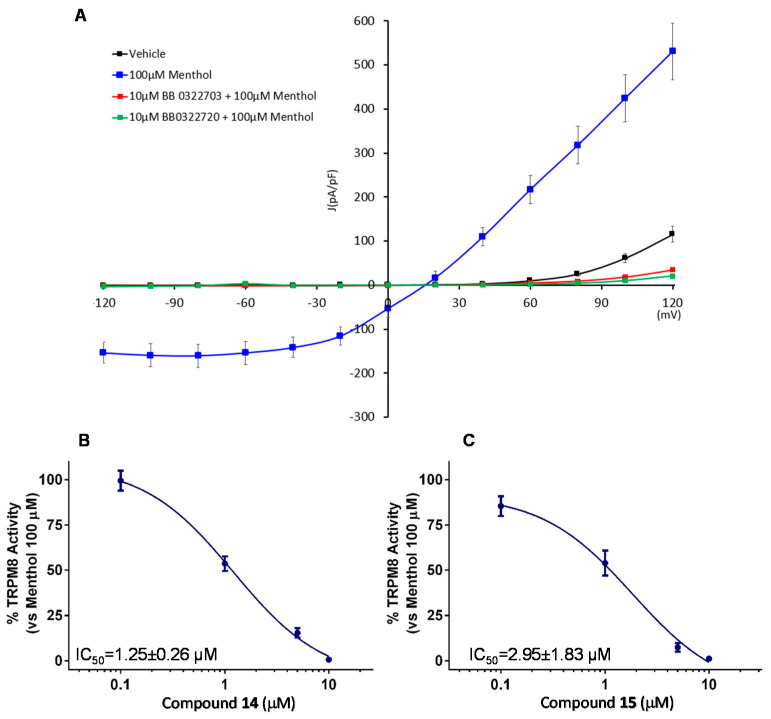
Compounds **14** and **15** (**BB 0322703** and **BB 0322720**, respectively) blocks TRPM8-mediated responses evoked by menthol in rTRPM8-expressing HEK293 cells (**A**) Curves obtained after: exposure to vehicle solution (black trace) 100 μM menthol (blue trace), 100 μM menthol + 10 μM 14 (BB 0322703) (red trace), and 100 μM menthol + 10 μM 15 (BB 0322720) (green trace). Peak current data were expressed as pA/pF (to allow comparison among different size cells). Each point is the mean ± SEM of n = 15. (**B**) Dose-response curve for the inhibitory effect over the currents evoked by 100 μM menthol in HEK293 cells expressing TRPM8 by compound **15** (**BB 0322720**). (**C**) Dose-response curve for the inhibitory effect over the currents evoked by 100 μM menthol in HEK293 cells expressing TRPM8 by compound **14** (**BB 0322703**). The solid line represents fits of the experimental data to the following equation: Y = Bottom + (Top − Bottom)/(1 + 10^((LogEC50-X)^^×^^HillSlope)^), with the restriction of the minimum (Top = 100) IC*_50_* = 1.25 (1.00–1.56) μM with r*^2^* = 0.99 and Hill slope = −1.23 for **14** (**BB 0322703**) and IC_50_ = 2.95 (0.82–10.61) μM with r^2^ = 0.97 and Hill slope= −0.71 for **15** (**BB 0322720**) Each point is the mean ± SEM of n = 15.

Considering the potency, efficacy, and selectivity shown, further investigation about TRPM8 antagonistic activity of compounds **14** and **15** (**BB 0322703** and **BB 0322720**) were performed by patch-clamp protocols. This study confirmed the efficacy of the two compounds, administered at 10 μM, in abolishing the TRPM8 menthol-evoked currents, reducing the current density at a lower level than the vehicle (Figure 6A). Dose-response curves were then recorded to evaluate the compound relative potencies. As shown in Figure 6B,C, both compounds showed low micromolar inhibitory potencies, with compound **14** (**BB 0322703**) slightly more potent. For this reason, the compound was selected for the following in vivo studies. 

### 2.6. In Vivo Assays

**Figure 7:**
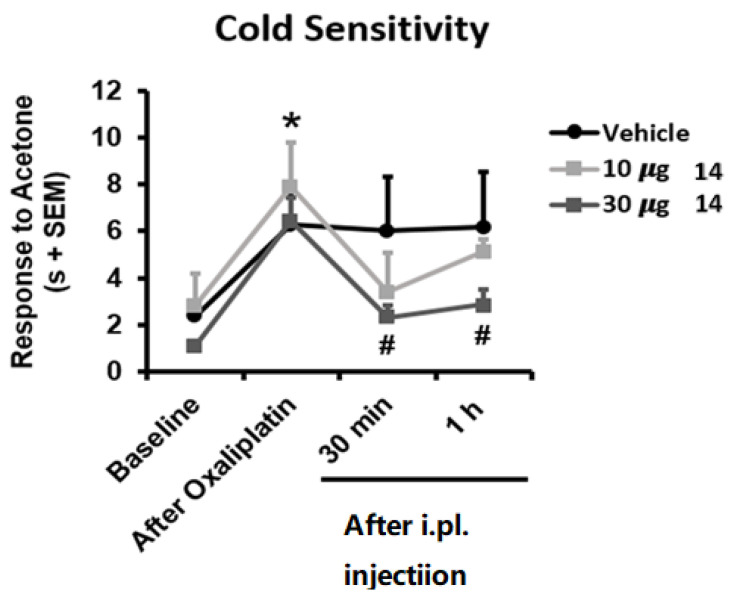
Reduced oxaliplatin (OXP)-induced cold allodynia by TRPM8 blocker **14** (**BB 0322703**). Mice were treated with three i.p. injections of OXP (6 mg/kg) or the vehicle (VH) on alternate days and on day 7 the cold allodynia behaviour was evaluated by the acetone test, before (baseline) and after OXP treatment. Time course effect of the TRPM8 blocker at 10 or 30 μg. Data are given as mean ± SEM (*n* = 6). one-way ANOVA with Tukey post hoc test. * *p* < 0.05 OXA vs. baseline. ^#^ *p* < 0.05 OXP vs. 30 μg **14** (**BB 0322703**).

TRPM8 antagonism is deeply related to analgesic in vivo effects and has been proposed as a suitable pharmacological strategy for the treatment of chronic pain, migraine, and painful syndromes [39,40,41]. TRPM8 antagonists are particularly known to counteract the chemotherapy-induced neuropathic pain evoked by oxaliplatin [24,27,28,29]. For these reasons we challenged compound **14** (**BB 0322703**) in vivo using the oxaliplatin-induced cold hypersensitivity assay. As shown in Figure 7, repeated oxaliplatin administration induced, as expected, cold hypersensitivity in the acetone drop test, reflected in longer duration of the response to acetone application to the hind paw (*p* < 0.05 vs. Baseline). Interestingly, mice receiving an intraplantar dose of 30 µg **14** (**BB 0322703**) showed significant inhibition of this cold hypersensitivity for at least 1 h (*p* < 0.05 vs. Oxaliplatin), whereas mice receiving 10 µg **14** (**BB 0322703**) or vehicle did not show significant modification of their nociceptive sensitivity at these time points. These results are in good accordance with previous reports concerning the use of TRPM8 antagonists as anti-allodynic agents in the oxaliplatin-induced allodynia model [28,29]. In particular, a correspondence between the in vitro potency and the effective in vivo dose is typical for TRPM8 modulators in the oxaliplatin-induced neuropathic pain model. Extremely in-vitro potent compounds are effective also at lower doses (10 mg) [28]. When in vitro potency is reduced [29], as also in the present case, in vivo efficacy decreases correspondingly. It is also worthy of note that non metabolically stable TRPM8 antagonists exhibit a short-acting effect (30 min, [28]) while metabolically stable analogs prolong the antiallodynic affect up to 1 h [29]. This is also the case of compound **14**, which for these reasons could be considered a promising hit-compound for the development of new chemotypes as TRPM8 antagonists and a pharmacological tool for the treatment of neuropathic pain. 

## 3. Discussion

The stepwise application of virtual and experimental screening to the selected molecular library allowed the identification of different compounds characterized by efficacy and potency in TRPM8 inhibition, confirming the robustness and the yield of this approach. In particular, two of these compounds showed low micromolar potencies when challenged in patch-clamp measurements, with compound **14** (**BB 0322703**) also exhibiting in vivo efficacy in a model of oxaliplatin-induced cold allodynia. It should be considered that this compound has been administered as a diastereoisomeric mixture of (R)-ethyl 2-((2-(((S)-1-phenylethyl)carbamoyl)phenyl)amino)propanoate and (R)-ethyl 2-((2-(((R)-1-phenylethyl)carbamoyl)phenyl)amino)propanoate. Molecular modelling studies suggest that the configuration S at the phenylethyl moiety represents the eutomer, since the other enantiomer was not retrieved among the top scoring compounds This prompts for further investigation about this chemotype, biological characterization of both the enantiomers would indeed help the discovery of more potent and promising derivatives.

On the other hand, **15** (**BB 0322720**), with its sulphonamide moiety, represents another interesting chemotype to be further investigated for hit-to-lead development of a new potent TRPM8 antagonist in consideration of its biological profile, chemical versatility, and metabolic stability. Moreover, selectivity studies of the reported compounds led to the identification of two dual-acting compounds, able to inhibit TRPM8 while activating TRPV1. Further studies involving these chemotypes would help in improving the knowledge about the structural discriminants for selectivity among these two channel subtypes. Additionally, considering the recent evidence about the analgesic and anticancer activity of both TRPV1 and TRPM8 modulators, it would be interesting to further explore the potential pharmacological activity of the dual-acting compounds **13** and **19** (**BB 0310244** and **BB 0310198**, respectively), in the search for more potent and effective pain-reliever and anticancer drug candidates. Nevertheless, further experiments have to be addressed in order to determine more accurate EC_50_ values for these two compounds.

## 4. Materials and Methods

### 4.1. Chemistry

NMR spectra were acquired on Bruker Avance 400 spectrometer (Bruker comporation, Billerica, MA, USA) at room temperature; the chemical shifts δ were measured in ppm with respect to solvent (1H: CDCl_3_, δ = 7.28 ppm; DMSO-*d*_6_: δ = 2.50 ppm; 13C: CDCl_3_, δ = 77.2 ppm; DMSO-*d*_6_: δ = 39.5 ppm). Splitting patterns are designated as s, singlet; d, doublet; t, triplet; q, quadruplet; quint, quintet; m, multiplet; dd, double doublet, br., broad. Coupling constants (*J*) are given in Hertz. The structures of synthesized compounds were elucidated with the aid of ^1^H, ^13^C spectroscopy. Low-resolution mass spectra were on Finnigan MAT mass spectrometer using electron ionization (direct inlet) and an ITD-700 detector with the ionizing electron energy being 70 eV and the mass range being *m*/*z* 35–400. The melting points (m.p.) were measured in open capillaries and presented without correction.

**Ethyl (R)-N-[2-{[(1-Phenylethyl)amino]carbonyl}phenyl]-2-aminopropionic acid (14)**

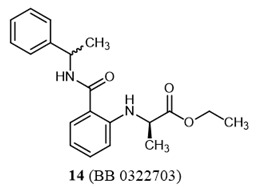



A mixture of a-methylbenzylamine (2.25 g, 2.1 mmol) in DMF (4 mL) and (R)-Ethyl-2-(2,4-dioxo-2H-3,1-benzoxazin-1(4H)-yl)propanoate [42] (3.26 g, 2.0 mmol) in DMF (10 mL) was stirred at 45–50 °C for 3 h. The reaction mixture was cooled to rt, diluted with cold water (100 mL), and then basified with 50% KOH to pH of 9. The resulting mixture was extracted with CH_2_Cl_2_, washed with water until the pH was ~7, and dried over anhydrous Na_2_SO_4_. The solvent was evaporated under reduced pressure. The crude product was purified by column chromatography on silica gel (eluent: ethyl acetate/petroleum ether = 1/10) to afford 14 (80%) as a yellowish oil (mixture of diastereomers D_#1_:D_#2_ = 1:1).

^1^H NMR (400 MHz, CDCl_3_, 25 °C), *δ*: 7.88–7.84 (m, 1H, NH), 7.41–7.34 (m, 5H, 5 × CH_Ar_), 7.30–7.23 (m, 2H, 2 × CH_Ar_), 6.64–6.60 (m, 2H, 2 × CH_Ar_), 6.58–6.55 (m, 1H, CHAr), 6.33–6.30 (m, 1H, NH), 5.33–5.25 (m, 1H, C*H*CH_3_), 4.29–4.08 (m, 4H, Ph*CH*CH_3_, *CH*_2_CH_3_), 1.60 (d, J = 6.9, 3H, *CH*CH_3_), 1.52 (dd, J_1_ = 7.0 Hz, J_2_ = 2.1 Hz, 3H, PhCHCH_3_), 1.25, 1.22 (both t, *J_D#1_* = 7.1, *J_D#2_* = 7.1, sum 3H, CH_2_*CH*_3_) ppm.

rt (D#1) = 1.376 min. LC–MS: *m*/*z* [M + H]^+^ = 341 Da.

rt (D#2) = 1.390 min. LC–MS: *m*/*z* [M + H]^+^ = 341 Da.

**3-(Benzenesulfonyl)-6-benzyl-1H-indole (15)**

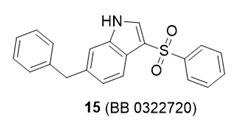



To a suspension of 540 mg (22.5 mmol) of sodium hydride in anhydrous dimethylformamide (40 mL), portions of 1.76 g (15.0 mmol) of 6-benzyl-1H-indole [43] were added, and the mixture was stirred at a temperature until the evolution of hydrogen completely ceases. Then diphenyl disulfide 3.6 g (16.5 mmol) was added portion wise and a mixture was stirred at room temperature for 18 h. The mixture is diluted with water (300 mL) and extracted with ether (3 × 45 mL). The extracts were combined and washed several times with water and dried over anhydrous Na_2_SO_4_. The solvent was evaporated. The crude was recrystallized from a mixture of toluene-hexane. Yield of 6-benzyl-3-(phenylthio)-1H-indole 2.95 g (95%).

6-Benzyl-3-(phenylthio)-1H-indole 2.90 g (9.2 mmol, 1 equiv) was dissolved in CH_2_Cl_2_ (250 mL) and to this solution m-chloroperbenzoic acid 5.55 g (32.2 mmol, 3.5 equiv) was added portion wise. The mixture was stirred at room temperature for 18 h. The reaction mixture was then poured into water (350 mL) and extracted with CH_2_Cl_2_ (3 × 50 mL). The extracts were combined and washed with water, an aqueous solution of sodium metabisulfate, sodium bicarbonate, and dried over Na_2_SO_4_. The crude was recrystallized from CH_2_Cl_2_. Yield 2.50 g (78%). M.p. 247–248 °C.

^1^H NMR (400 MHz, CDCl_3_), δ: 12.29 (1H, c, NH), 8.00 (s, 1H), 7.57 (d, *J* = 8.1 Hz, 1H), 7.32–7.26 (m, 6H), 7.25–7.18 (m, 4H), 7.15 (dd, *J* = 8.4, 6.0 Hz, 1H), 6.55–6.47 (m, 1H), 4.11 (s, 2H) ppm.

rt = 1.420 min. Purity = 100%. LC–MS: *m*/*z* [M + H]+ = 348 Da.

### 4.2. In Vitro Biological Assays

#### 4.2.1. Cell Cultures

Cells, HEK-293 cells stably expressing rTRPM8 or hTRPV1, were cultured in a monolayer in Earle’s minimum essential medium with Earle’s salts supplemented with 10% fetal calf serum, 1% nonessential amino acids, 2 mM L-glutamine, 100 µg streptomycin/mL, 100 U penicillin/mL, and 0.4 µg/mL puromycin (referred to as Puro-EMEM) and kept at 37 °C in a humidified atmosphere of 5% CO_2_ in a suitable incubator (STERI-CyCLE CO_2_ Incubator Hepa Class 100, Thermo Electrón Corporation, Waltham, MA, USA). Cells were stored under liquid nitrogen and used for 15 generations from unfreezing.

For fluorometric experiments, HEK-293 cells stably expressing rTRPM8 or hTRPV1 were detached by means of Trypsin/EDTA solution, resuspended in DMEM-10%FCS and seeded at a concentration of 2 × 10^4^ cells/mL.

#### 4.2.2. Fluorometric Assays

To measure the effectiveness of the compounds against TRPM8 activity we have used microfluorometry-based calcium flux assays with Fluo-4 NW Ca^2+^ dye and fluorescence as described previously [44]. Briefly human embryonic kidney cell line (HEK) stably transfected with rTRPM8 or hTRPV1 were seeded in 96-well plates at a cell density of 30,000 cells. After 2 days the medium was replaced with 100 μL of the dye loading solution Fluo-4 NW supplemented with probenecid 2.5 mM and incubated 1 h at 37 °C. The TRP ion channel activity was measured using POLASTAR plate reader (BMG Labtech, Ortenberg, Germany) setting the excitation wavelength at 485 nm and emission wavelength at 520 nm. The baseline fluorescence was recorded for 3 cycles before the addition of the vehicle, compound at different concentrations, and the antagonist, 10 μM AMTB for TRPM8 and Ruthenium red for TRPV1. Fluorescence intensity was recorded during 7 more cycles and agonist 100 μM menthol for TRPM8 or 10 μM capsaicin for TRPV1 was added. Fluorescence intensity was recorded during 10 more cycles. 

Data analysis: The Z-factor was calculated in each assay using the following equation: (3 × (SDmax + SDmin))/(Mean max − Mean min). In all the experiments, the Z-factor was ≥0.5. The effect of the compounds against TRPM8 activity was determined by normalizing their effect to the maximum fluorescence observed with the application of 100 μM menthol. A decrease in menthol signal was expressed as a percentage of inhibition (%). All data are expressed as the mean ± standard deviation (SD). Data are expressed as the concentration exerting a half-maximal inhibition of agonist-induced (Ca^2+^)^i^ elevation (IC_50_), which was calculated again using GraphPad Prism^®^ software (GraphPad Prism 5, Graphpad Software, San Diego, CA, USA). The equation used was Y = Bottom + (Top − Bottom)/(1 + 10^((LogEC50-X)^ ^× HillSlope)^), with the restriction of the minimum (Bottom = 0). All determinations were performed in triplicate (*n* = 3) in 3 independent experiments (*n*= 3).

#### 4.2.3. Patch-Clamp Experiments

Whole-cell patch-clamp recordings from HEK-rTRPM8 cells were carried out 2 days after seeding on 12-mm Ø glass coverslips treated with poly-L-lysine solution (Sigma Aldrich, Madrid, Spain) [30]; the intracellular pipette solution contained (in mM) 150 NaCl, 5 EGTA, 3 MgCl_2,_ and 10 HEPES, adjusted to pH 7.2 with NaOH, and the extracellular solution contained (in mM) 150 NaCl, 6 CsCl, 1.5 CaCl_2_, 1 MgCl_2_, 10 D-glucose and 10 HEPES, adjusted to pH 7.4 with NaOH. The TRPM8 activity was measured by the application of two pulses of 100 μM menthol in a time interval of 2 min and 30-s perfusion of the different compound concentrations before the second menthol pulse. 

Menthol (100 µM) or compounds (0.1–10 µM; 2 min perfusion) were applied directly onto the cell under investigation by means of a multibarrel concentration-clamp device coupled to an electronically driven miniature solenoid valves under the control of PatchMaster software (HEKA Electronics, Lambrecht, Germany). 

Data were sampled at 10 kHz (EPC10 amplifier with PatchMaster 2.53 software, HEKA Electronics, Lambrecht, Germany) and low-pass filtered at 3 kHz for analysis (PatchMaster 2.53 and GraphPad Prism 5). The series resistance was <10 MΩ and to minimize voltage errors was compensated to 60–80%. All measurements were performed at 24–25 °C. Cell capacitance was measured and used to estimate the current density (J, pA/pF).

For the whole-cell patch-clamp experiments, results are expressed as the percentage of the remaining activation of the TRPM8 channel. This is calculated by normalizing the ratio (p2/p1) of testing conditions to the ratio (p2/p1) of the control condition. The analysis of the data was performed by GraphPad 6.0, the ordinary one-way ANOVA analysis followed by the post hoc Bonferroni test established multiple comparisons, and the ROUT method (Q = 10%) identified data outliers. A non-linear regression curve and IC50 were obtained by the representation of log (inhibitor) vs. response. the equation used was Y = Bottom + (Top − Bottom)/(1 + 10^((LogEC50-X) × HillSlope)^), with the restriction of the maximum (Top = 100). Paired or unpaired Student’s *t*-tests were also used for data comparison between two groups. All data are expressed as the mean ± standard error of the mean (SEM) (*n* = 12–15).

### 4.3. In Vivo Studies

#### 4.3.1. Animals

C57BL/6JRccHSd female mice (10–15 week-old, ~20 g) (Harlan, The Netherlands) were used for the oxaliplatin-induced neuropathic pain study. Care was taken to minimize the number of animals used and the pain and stress they experienced. All experimental procedures were approved by the Animal Care and Use Committees of Universidad Miguel Hernández and the regional government and were conducted according to the ethical principles of the International Association for the Study of Pain (IASP) for the evaluation of pain in conscious animals [45], the European Parliament and the Council Directive (2010/63/EU) and the Spanish law (RD 53/2013). Housing conditions were maintained at 21 ± 1 °C and 55 ± 15% relative humidity in a controlled light/dark cycle (light on between 8:00 a.m. and 8:00 p.m.). Animals had free access to food and water except during the habituation or the evaluation of nociceptive sensitivity. All parts of the study concerning animal care were performed under veterinary control.

#### 4.3.2. Drugs

Compound **14** (**BB 0322703**) was dissolved in 2.5% dimethyl sulfoxide (Merck, Darmstadt, Germany) and 2.5% cremophor EL (Merck) diluted in saline. Compound **14** (**BB 0322703**) was prepared at 1.2 or 0.4 mg/mL and intraplantar injected in a 25 μL volume. Oxaliplatin was obtained from Tocris (ref#2623, Tocris, Bristol, UK).

#### 4.3.3. Oxaliplatin-Induced Neuropathic Pain

Oxaliplatin-induced neuropathic pain was induced by repeated oxaliplatin administration (Cat. No. 2623, Tocris, Bristol, UK). Oxaliplatin was freshly prepared every day of administration. It was dissolved in water with 5% dextrose after gentle warming, and it was intraperitoneally injected every other day for 5 days at a 6 mg/kg dose. Nociceptive sensitivity was assessed before oxaliplatin treatment and 3 days after the last oxaliplatin administration.

#### 4.3.4. Cold Sensitivity

Cold chemical thermal sensitivity was assessed using the acetone drop method as previously described. Briefly, mice were first handled and habituated to the experimenter before the experiments. To measure acetone sensitivity, mice were placed over a metal grid and allowed to habituate for approximately 1 h. Freshly dispensed acetone drops (20 μL) were applied gently onto the mid-plantar surface of the right hind paw. Paw licking, attending, dragging, or lifting were considered nociceptive-like responses. The responses were measured for 1 min with a digital stopwatch. For each measurement, the paw was sampled three times and the mean was calculated. The interval between each application of acetone was at least 3 min.

#### 4.3.5. Statistical Analysis

Behavioral data were analyzed with a repeated-measures ANOVA followed by a post-hoc Tukey test (GraphPad Prism 7.04, GraphPad Software Inc.). *p*-values lower than 0.05 were considered statistically significant.

### 4.4. In Silico-Studies

#### 4.4.1. Models Building

The hTRPM8 protein sequence Q7Z2W7 was downloaded from the Universal Protein Resource (UniProt) [46] and template Cryo-EM structures of TRPM8 cold receptor in complex with AMTB (PDB ID: 6O6R) and TC-I 2014 (PDB ID: 6O72) were downloaded from the Protein Data Bank [2]. The region of our interest that comprised domains S1 to TRP (residues 723–1013) was aligned using ClustalW [47] to *Parus major* TRPM8 and modelled by homology with the two experimentally solved template structures. It is worth noting that *Parus major and human* TRPM8 share a high degree of similarity (Identity: 82%; Similarity: 92%; Conservation: 91%), analogously to *Ficedula albicollis* [25], *Mus musculus* and *Rattus norvegicus* [26]. 

The energy-based method of Prime [48,49] was used to build two models of hTRPM8_723–1013_ tetramer: in complex with AMTB (A_hTRPM8_723–1013_—template 6O6R) and in complex with TC-I 2014 (T_hTRPM8_723–1013_—template 6O72). Rotamers of conserved residues were retained and all side chains were optimized. The two models and the library to screen were then prepared, as previously reported [50], for the subsequent studies, using the Schrödinger Protein Preparation Wizard [51] and the ligprep utility [52]. Docking calculations were carried on by means of Glide [53,54,55] using a target ensemble composed of the two previously prepared protein structures, i.e., AMTB/hTRPM8_723–1013_ and TC-I/hTRPM8_723–1013_.

The docking space was defined as a (35 Å)^3^ cube centered on the bound pose of AMTB for A_hTRPM8_723–1013_ and of TC-I 2014 for T_hTRPM8_723–1013_; the ligands diameter midpoints were required to dock into a smaller, nested (20 Å)^3^ cube. No constraint was applied during docking calculations, nonpolar ligand atoms (partial charge < 0.25) were scaled by a 0.80 factor, and protein hydroxyl and thiol groups were allowed to rotate. Molecular docking simulations were performed in a stepwise manner using Glide SP and Glide XP. The library to screen (2543 compounds; 4721 after ligand preparation) was submitted to Glide SP docking and all docking poses scoring worse than −4 were ruled out, while those scoring better than −4 were advanced to the subsequent step where their docking poses were refined, minimized and re-scored using Glide XP. For each ligand, the best scoring pose only was finally retrieved, regardless of the target it was retrieved by, as in previously reported studies [56,57]. 

#### 4.4.2. Molecular Dynamics Simulations of **14** (**BB 0322703**) and **15** (**BB 0322720**) in Complex with hTRPM8_723–1013_

Predicted bound conformations of **14** (**BB 0322703**) and **15** (**BB 0322720**) in complex with T_hTRPM8_723–1013_ were submitted to molecular dynamics MD simulations in order to investigate the most important inhibitor/ligand interactions. The simulations were set up and run using Desmond [58]. The simulated environments were inserted into a POPC bilayer, based on the coordinates (ID: 6O72) downloaded from the OPM database [59]. 

OPLS-2005 [60] was used as a force field and solvation was treated explicitly by the TIP3P water model [61]. The system was neutralized by Na^+^ and Cl^−^ ions that were added to the final concentration of 0.15 M. Protein/membrane systems were submitted to the standard equilibration protocol for membrane proteins distributed with Desmond. Systems were finally submitted to 120 ns long MD simulations at a temperature of 300 K in the isothermal−isobaric ensemble using a Nose−Hoover chain thermostat and a Martyna−Tobias−Klein barostat. Backbone atoms were constrained during the simulation (1 kcal/mol). Trajectory analyses were performed using the Desmond simulation event analysis and the simulation interaction diagram tools.

## Figures and Tables

**Table 1:** Compounds selected for experimental screening.

Compound	XP GlideScore	VS Rank ^a^
**1 (BB 0220221)**	−9.315	57
**2 (BB 0301246)**	−9.556	34
**3 (BB 0301259)**	−9.621	29
**4 (BB 0301261)**	−9.382	49
**5 (BB 0304240)**	−9.019	85
**6 (BB 0304398)**	−9.432	39
**7 (BB 0304425)**	−9.245	64
**8 (BB 0305409)**	−9.786	22
**9 (BB 0305411)**	−9.004	87
**10 (BB 0305430)**	−9.528	35
**11 (BB 0310197)**	−9.400	43
**12 (BB 0310217)**	−9.349	51
**13 (BB 0310244)**	−9.589	31
**14 (BB 0322703)**	−9.242	66
**15 (BB 0322720)**	−9.063	81
**16 (BB 0323219)**	−9,092	79
**17 (BB 0323225)**	−9.106	77
**18 (BB 0301235)**	−10.104	14
**19 (BB 0310198)**	−10.235	10
**20 (BB 0310207)**	−10.694	6
**21 (BB 0237332)**	−8.964	90

^a^ Compounds ranking by XP GlideScore. Full ranking of top scoring 100 compounds, together with their structures and XP GlideScore values, is given in supporting information.

**Table 2:** IC_50s_ and efficacy for the selected compounds. Results are given as mean ± SD of at least three independent measurements.

Compound	IC_50_ (µM)	Efficacy ^a^
**1 (BB 0220221)**	10.21 ± 1.31	85
**5 (BB 0304240)**	11.10 ± 1.32	95
**13 (BB 0310244)**	1.01 ± 0.51	90
**14 (BB 0322703)**	0.25 ± 0.15	100
**15 (BB 0322720)**	0.22 ± 0.10	100
**19 (BB 0310198)**	5.52 ± 1.45	90
**AMTB**	7.15 ± 1.24	100

^a^ Expressed as a percentage of inhibition of menthol evoked currents at the maximum concentration used (50 µM).

## Data Availability

The data presented in this study are available in the supplementary information file. Any other raw data can be requested to the corresponding authors.

## Supplementary Materials

The following supporting information can be downloaded at: https://www.mdpi.com/article/10.3390/ijms23042070/s1.
