# Supplementary material for: In Vitro and In Vivo Pharmacological Characterization of a Novel TRPM8 Inhibitor Chemotype Identified by Small-Scale Preclinical Screening
Source: Int J Mol Sci. 2022 Feb 13;23(4):2070. doi: 10.3390/ijms23042070 (PMC8877448; doi:10.3390/ijms23042070)
Supplement: Supplementary file 1 [file ijms-23-02070-s001.zip › Table S1.pdf]

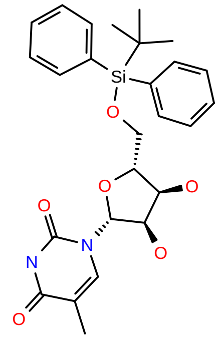

title	BB 0294578
rank	1
score	-11.982

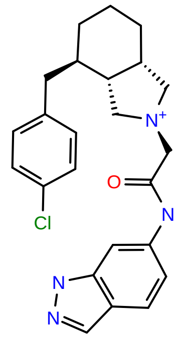

title	BB 0301275
rank	2
score	-10.969

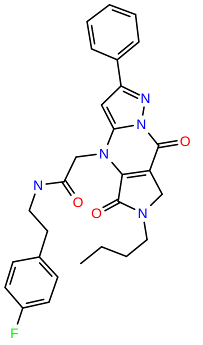

title	BB 0310261
rank	3
score	-10.912

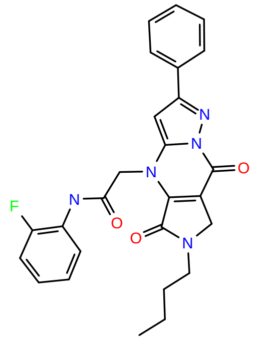

title	BB 0310265
rank	4
score	-10.842

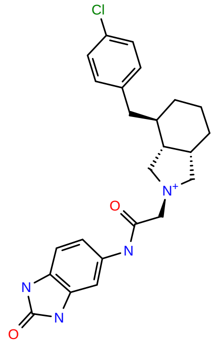

title	BB 0301273
rank	5
score	-10.811

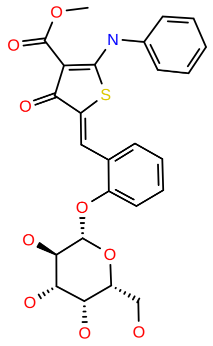

title	BB 0310207
rank	6
score	-10.694

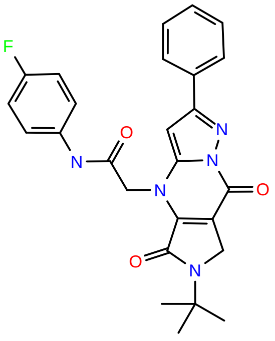

title	BB 0310262
rank	7
score	-10.692

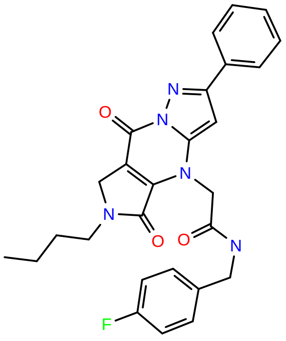

title	BB 0310260
rank	8
score	-10.526

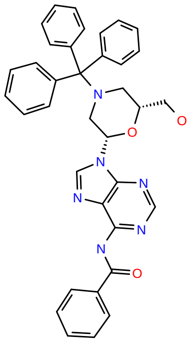

title	BB 0300977
rank	9
score	-10.364

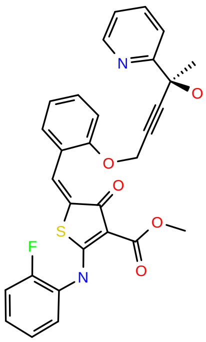

title	BB 0310198
rank	10
score	-10.235

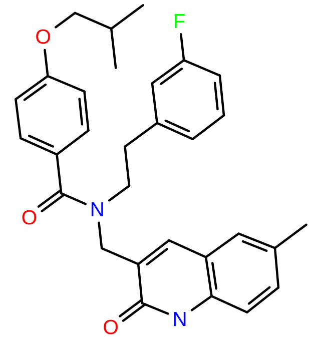

title	BB 0263220
rank	11
score	-10.184

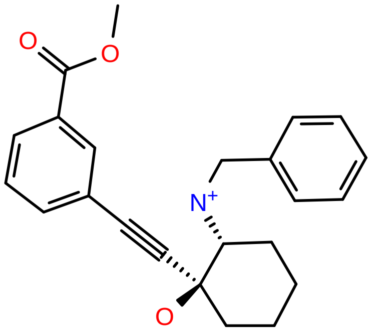

title	BB 0310810
rank	12
score	-10.114

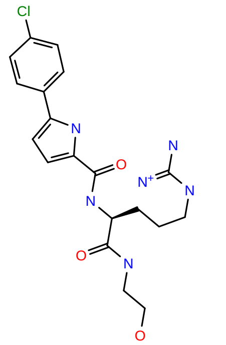

title	BB 0304052
rank	13
score	-10.111

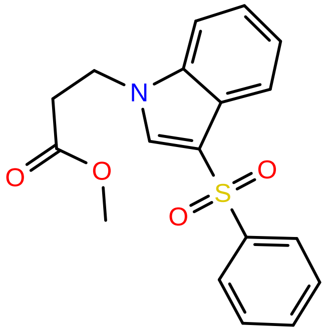

title	BB 0301235
rank	14
score	-10.104

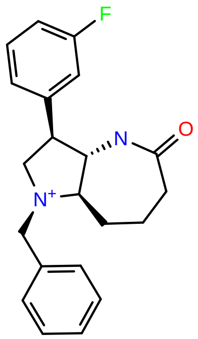

title	BB 0263371
rank	15
score	-9.956

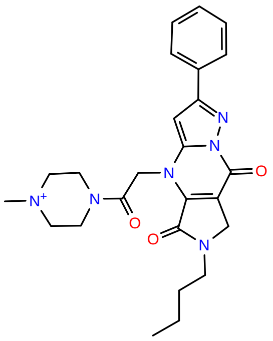

title	BB 0310264
rank	16
score	-9.938

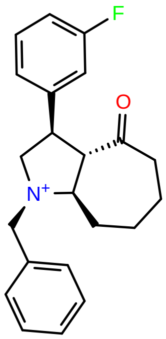

title	BB 0265811
rank	17
score	-9.912

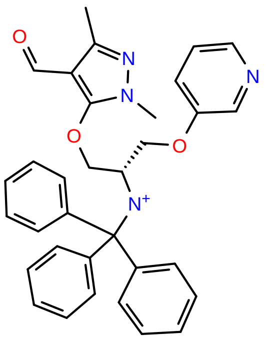

title	BB 0304784
rank	18
score	-9.883

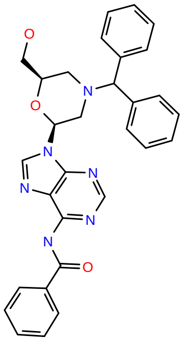

title	BB 0323026
rank	19
score	-9.862

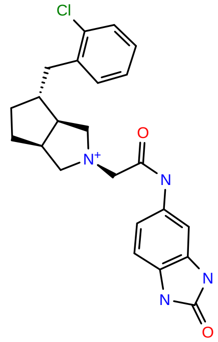

title	BB 0301276
rank	20
score	-9.835

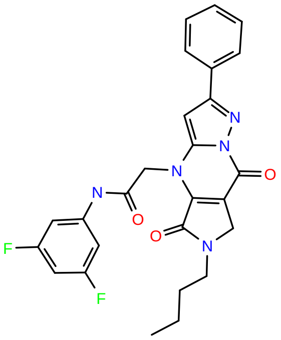

title	BB 0310274
rank	21
score	-9.808

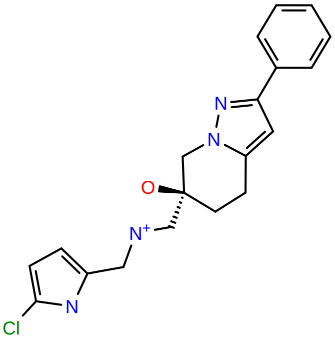

title	BB 0305409
rank	22
score	-9.786

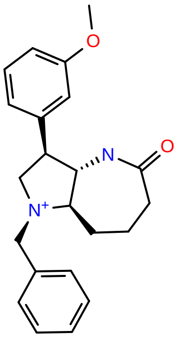

title	BB 0263370
rank	23
score	-9.785

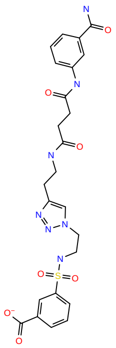

title	BB 0304363
rank	24
score	-9.771

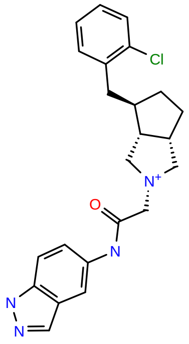

title	BB 0301277
rank	25
score	-9.698

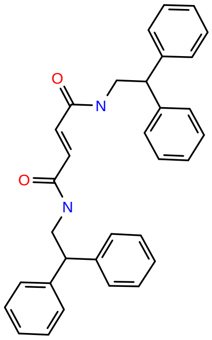

title	BB 0266576
rank	26
score	-9.656

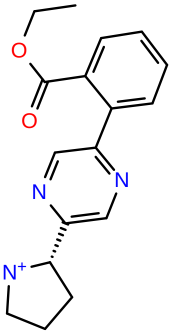

title	BB 0304027
rank	27
score	-9.648

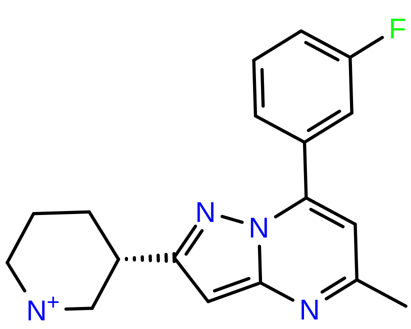

title	BB 0322789
rank	28
score	-9.632

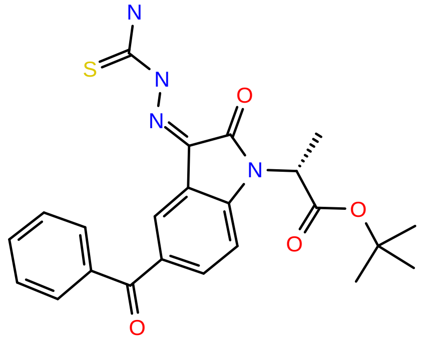

title	BB 0301259
rank	29
score	-9.621

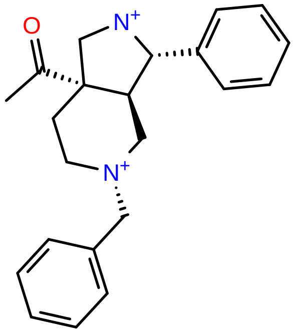

title	BB 0263364
rank	30
score	-9.608

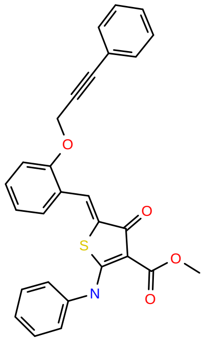

title	BB 0310244
rank	31
score	-9.589

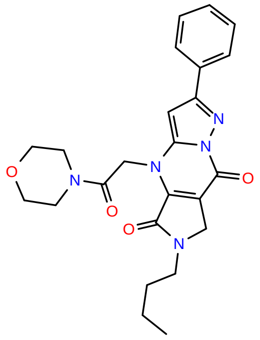

title	BB 0310273
rank	32
score	-9.564

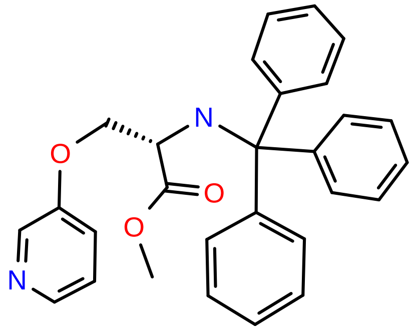

title	BB 0304782
rank	33
score	-9.563

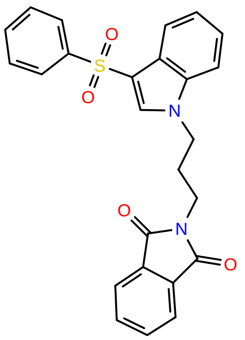

title	BB 0301246
rank	34
score	-9.556

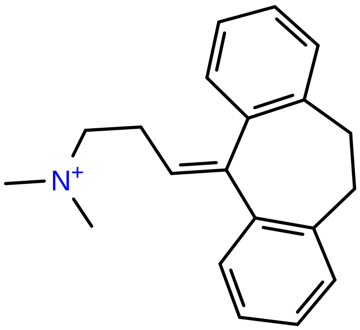

title	BB 0305430
rank	35
score	-9.528

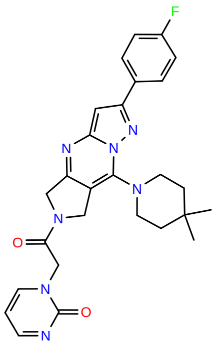

title	BB 0323120
rank	36
score	-9.478

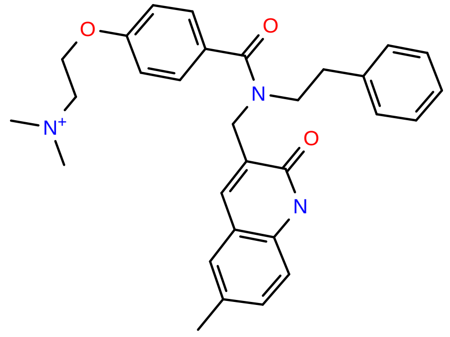

title	BB 0263224
rank	37
score	-9.455

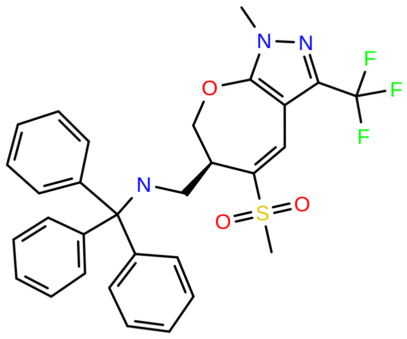

title	BB 0310327
rank	38
score	-9.449

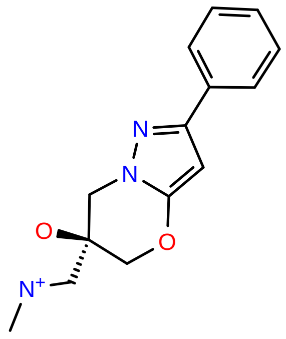

title	BB 0304398
rank	39
score	-9.432

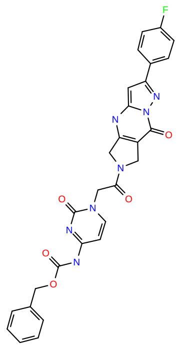

title	BB 0323151
rank	40
score	-9.425

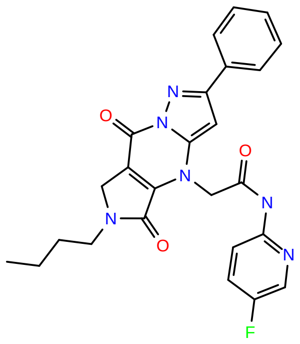

title	BB 0310275
rank	41
score	-9.41

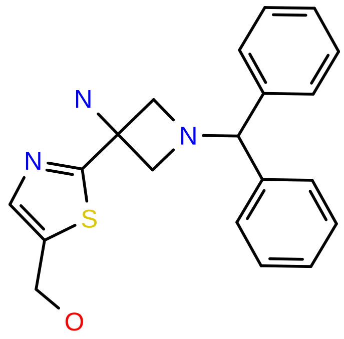

title	BB 0310057
rank	42
score	-9.408

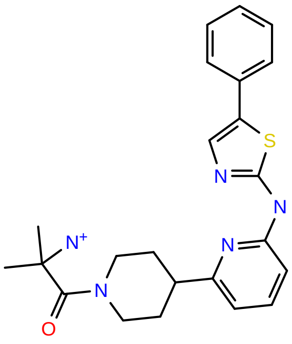

title	BB 0310197
rank	43
score	-9.4

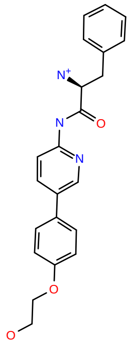

title	BB 0305926
rank	44
score	-9.399

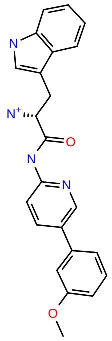

title	BB 0305921
rank	45
score	-9.392

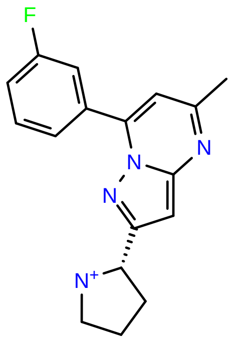

title	BB 0322728
rank	46
score	-9.391

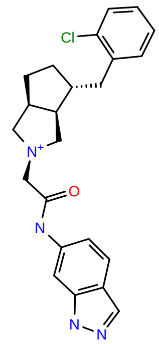

title	BB 0301153
rank	47
score	-9.386

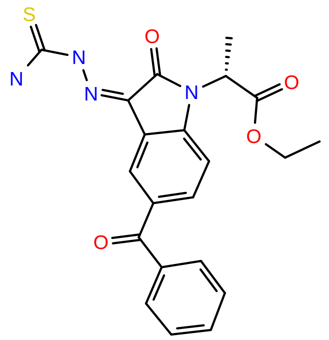

title	BB 0301261
rank	49
score	-9.382

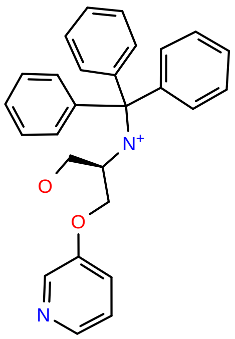

title	BB 0304783
rank	48
score	-9.382

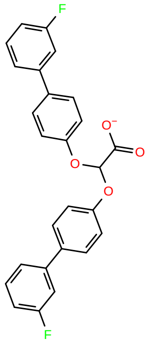

title	BB 0266665
rank	50
score	-9.372

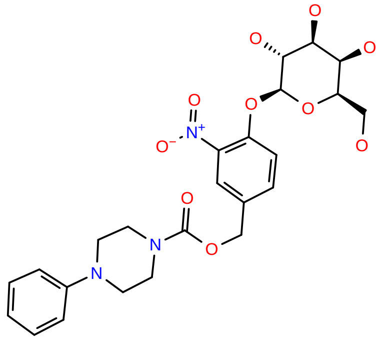

title	BB 0310217
rank	51
score	-9.349

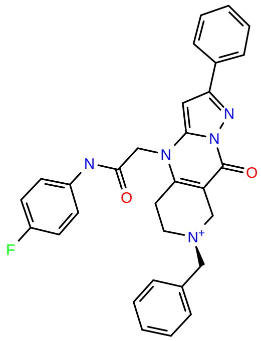

title	BB 0310257
rank	52
score	-9.337

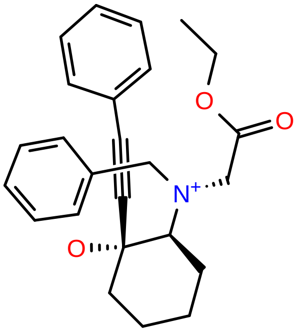

title	BB 0310743
rank	53
score	-9.335

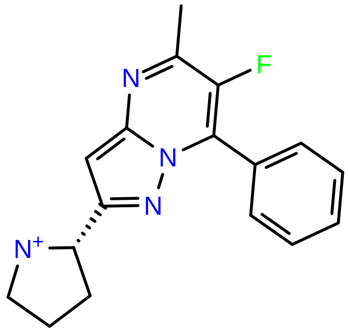

title	BB 0322741
rank	54
score	-9.335

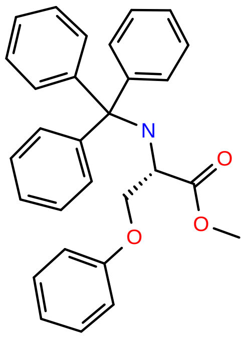

title	BB 0304187
rank	55
score	-9.333

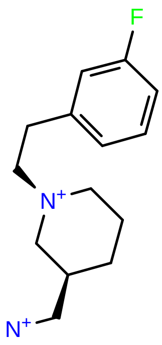

title	BB 0220221
rank	57
score	-9.315

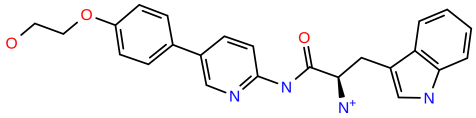

title	BB 0305927
rank	56
score	-9.315

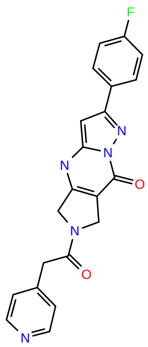

title	BB 0310322
rank	58
score	-9.312

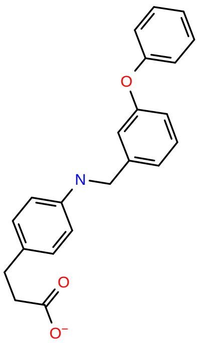

title	BB 0304209
rank	59
score	-9.298

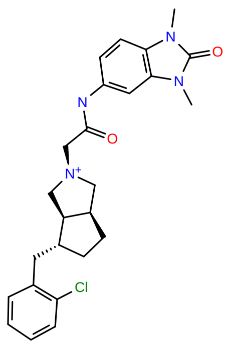

title	BB 0301274
rank	60
score	-9.298

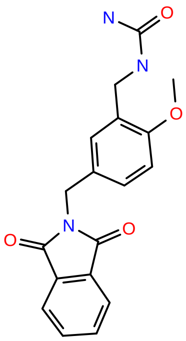

title	BB 0266686
rank	61
score	-9.293

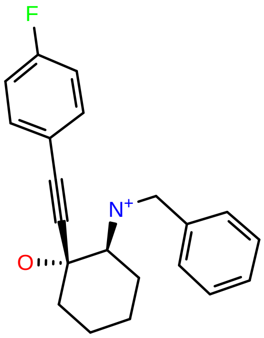

title	BB 0323009
rank	62
score	-9.287

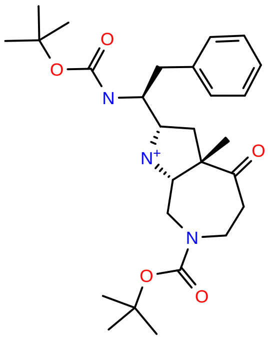

title	BB 0304418
rank	63
score	-9.279

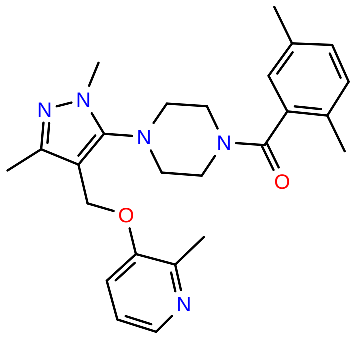

title	BB 0304425
rank	64
score	-9.245

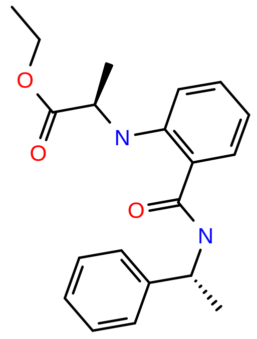

title	BB 0322703
rank	66
score	-9.242

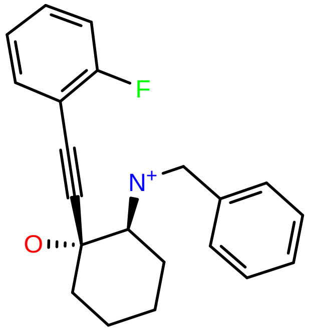

title	BB 0323007
rank	65
score	-9.242

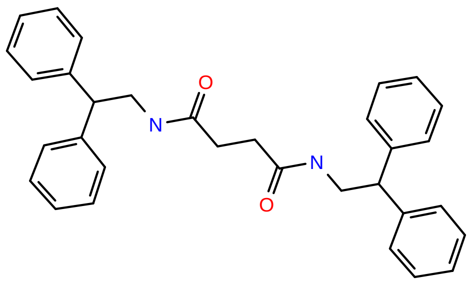

title	BB 0266577
rank	67
score	-9.226

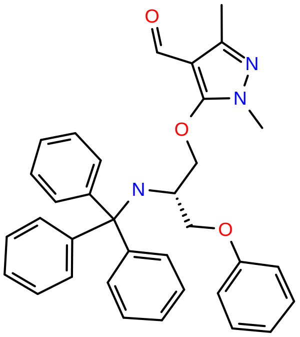

title	BB 0304208
rank	68
score	-9.212

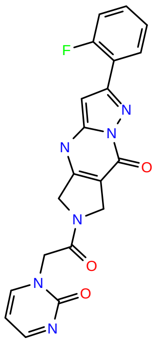

title	BB 0323132
rank	69
score	-9.195

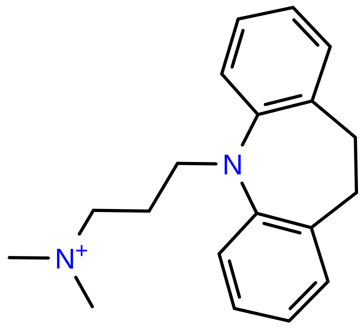

title	BB 0304427
rank	70
score	-9.165

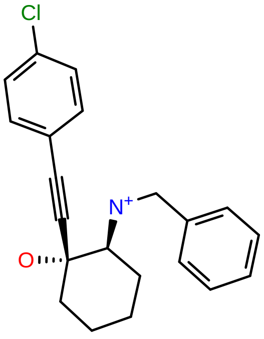

title	BB 0323018
rank	71
score	-9.157

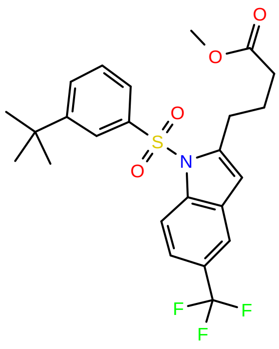

title	BB 0305728
rank	72
score	-9.153

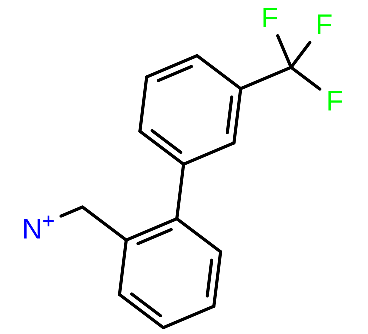

title	BB 0254060
rank	73
score	-9.121

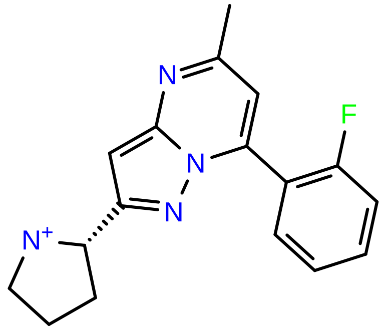

title	BB 0322732
rank	74
score	-9.116

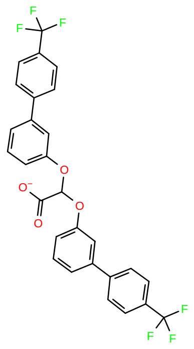

title	BB 0269314
rank	75
score	-9.114

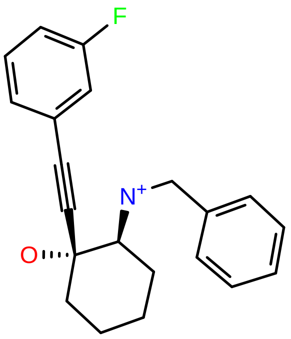

title	BB 0323008
rank	76
score	-9.108

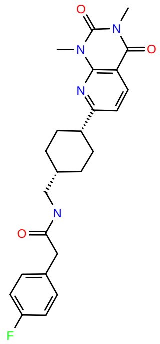

title	BB 0323225
rank	77
score	-9.106

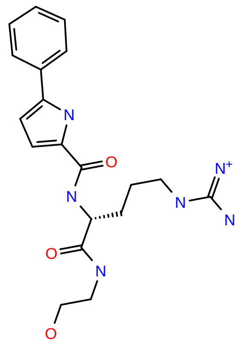

title	BB 0265849
rank	78
score	-9.106

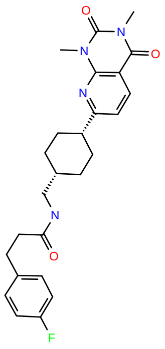

title	BB 0323219
rank	79
score	-9.092

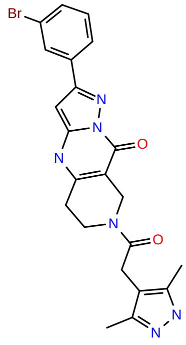

title	BB 0323137
rank	80
score	-9.076

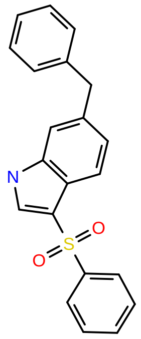

title	BB 0322720
rank	81
score	-9.063

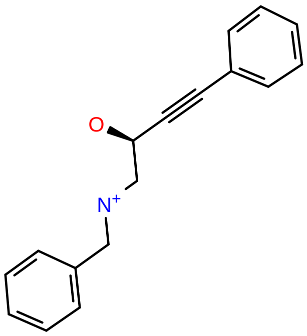

title	BB 0305187
rank	82
score	-9.056

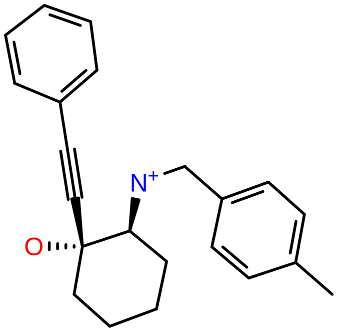

title	BB 0310738
rank	83
score	-9.056

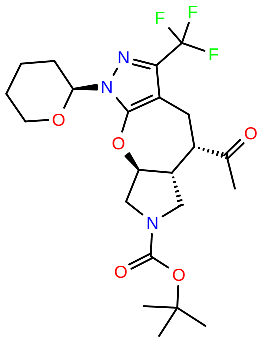

title	BB 0310230
rank	84
score	-9.034

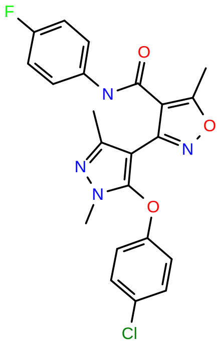

title	BB 0304240
rank	85
score	-9.019

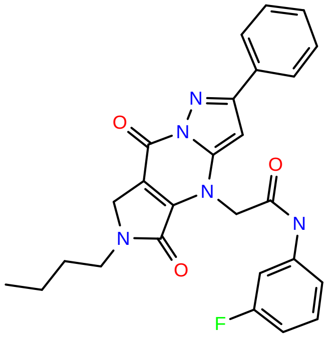

title	BB 0310258
rank	86
score	-9.013

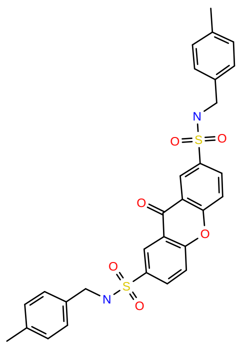

title	BB 0305411
rank	87
score	-9.004

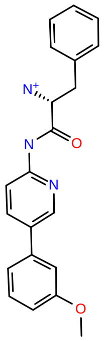

title	BB 0305924
rank	88
score	-8.983

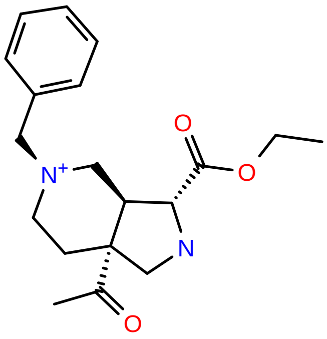

title	BB 0322374
rank	89
score	-8.968

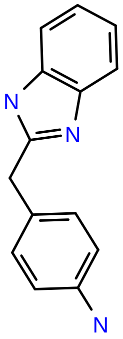

title	BB 0237332
rank	90
score	-8.964

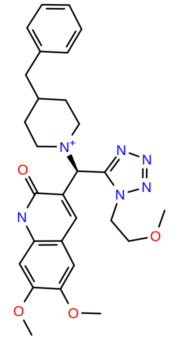

title	BB 0268594
rank	91
score	-8.956

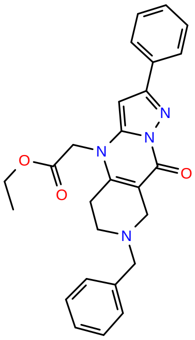

title	BB 0310254
rank	92
score	-8.955

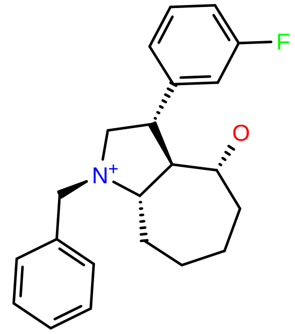

title	BB 0267167
rank	93
score	-8.954

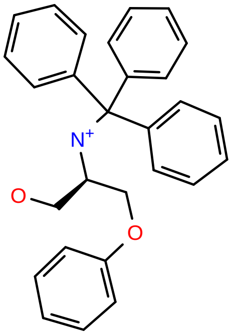

title	BB 0304190
rank	94
score	-8.95

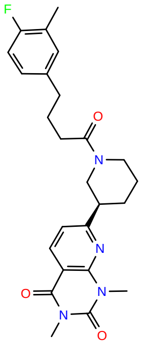

title	BB 0323233
rank	95
score	-8.936

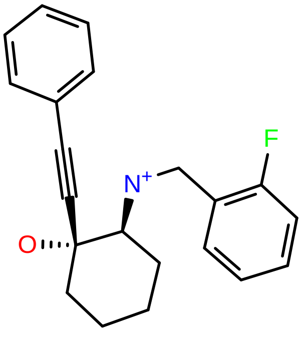

title	BB 0305178
rank	96
score	-8.935

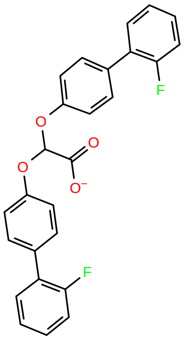

title	BB 0266666
rank	97
score	-8.92

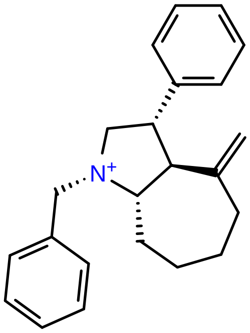

title	BB 0267166
rank	98
score	-8.919

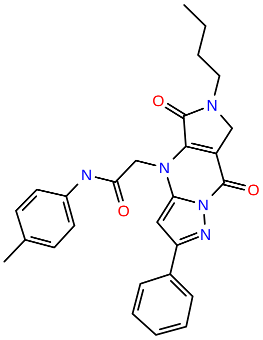

title	BB 0310263
rank	99
score	-8.916

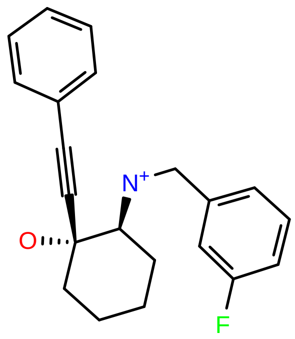

title	BB 0305171
rank	100
score	-8.903
